# Commentary on “Setting up a COVID-19 care facility at a prison: An experience from Pakistan”

**DOI:** 10.1016/j.amsu.2020.09.018

**Published:** 2020-09-10

**Authors:** Syed Ghulam Sarwar Shah, Sayema Awais, Sayed Fida Hussain Shah

**Affiliations:** a NIHR Oxford Biomedical Research Centre, Oxford University Hospitals NHS Foundation Trust, John Radcliffe Hospital, Headington Way, Headington, Oxford, OX3 9DU, England, UK; b Health Services Academy, Chak Shahzad, Islamabad, 44000, Pakistan; c Department of Surgery, Minimal Invasive Surgical Centre, Bilawal Medical College, Liaquat University of Medical and Health Sciences, Jamshoro, 76090, Sindh, Pakistan

Ayyaz et al. [1] have reported the establishment of a COVID-19 care facility for inmates in a jail in Pakistan. This study is a unique study amongst numerous studies that have been published so far on the COVID-19 pandemic because it is about the testing of COVID-19 and providing healthcare to prisoners who are one of the most vulnerable groups to COVID-19 because they live in incarcerated settings [2]. Another unique characteristic of the study is that it has been undertaken in a higher-risk setting i.e. a prison in a resource constrained country i.e. Pakistan - a lower middle income country (LMIC).

Ayyaz et al. have reported various important actions that were taken whilst setting up the COVID-19 facility in the prison such as temporary stoppage of the admissions of new prisoners, testing of inmates and staff for COVID-19, caring for prisoners who were COVID-19 positive and taking preventative measures such as providing sanitizers for handwashing and contact tracing to prevent the spread of coronavirus infection amongst prisoners, staff and visitors to the prison and providing healthcare to the prisoners who had COVID-19 [1]. Besides successful outcomes such as a limited morbidity and no mortality due to COVID-19 in the prison, Ayyaz et al. have highlighted some key issues such as the involvement of all key stakeholders, the need for proper preparation and coordination, and encountering different challenges and problems whilst undertaking this important and difficult healthcare project during the COVID-19 pandemic [1]. However, they have neither described the problems and challenges nor discussed how these were overcome. Describing these important issues would have been helpful in understanding the nature of challenges and taking appropriate actions whilst planning for and setting up of similar COVID-19 care facilities elsewhere in response to the COVID-19 pandemic.

The COVID-19 pandemic has resulted in little more than 23 million confirmed cases and about 0.8 million deaths globally, as of 24th August 2020 [3]. The COVID-19 has directly or indirectly affected almost every individual albeit at a varying level. However, some people are more vulnerable to COVID-19 than others such as people in care homes [4] and prisoners in jails [5]. These places are often overcrowded, more likely hotbeds for the spread of communicable diseases and mostly have limited healthcare resources and facilities [6,7].

It is therefore imperative to prioritise the most vulnerable people in COVID-19 testing and healthcare provision during the COVID-19 pandemic. Besides prisoners, there are many other vulnerable people such as patients with longterm conditions and elderly in care homes, people in shelter homes / houses, orphanages and similar other dwellings, mental health patients in psychiatric centres and mental health institutions and hospitals, and refugees and displaced people in camps and detention centres. These disadvantaged and vulnerable people must need special attention and priority in testing for COVID-19 and providing them necessary healthcare onsite is also imperative.

## Provenance and peer review

Not commissioned, Editor reviewed.

## Funding

No funding

## Ethical approval

Not applicable because this is a commentary on an article published in the Ann Med Surg.

## Consent

Not applicable because this is a commentary on an article published in the Ann Med Surg.

## Registration of research studies

1. Name of the registry: N/A.

2. Unique Identifying number or registration ID: N/A.

3. Hyperlink to your specific registration (must be publicly accessible and will be checked): N/A.

## Author contribution

Study conception, planning and design; All authors.

Literature identification and review: S.G.S. Shah.

Manuscript drafting: S.G.S. Shah.

Manuscript review and intellectual input: S.F.H. Shah and S. Awais.

## Guarantor

The Guarantor is the one or more people who accept full responsibility for the work and/or the conduct of the study, had access to the data, and controlled the decision to publish. Please note that providing a guarantor is compulsory.

Syed Ghulam Sarwar Shah (S.G.S. Shah)

## Declaration of competing interest

Authors declared no conflict of interest.
